# Non-suicidal self-injury among youth students during COVID-19 pandemic: the role of psychological factors in Jingzhou, China

**DOI:** 10.3389/fpsyt.2024.1446727

**Published:** 2024-08-21

**Authors:** Lie Zhou, Ye Yu, Bo Liu, Li-Fang Zhou, Juan Sheng, Xin-Feng Zhang, Xiao-Peng Deng, Mao-Sheng Ran

**Affiliations:** ^1^ Mental Health Center of Yangtze University, Jingzhou, Hubei, China; ^2^ Mental Health Institute of Yangtze University, Jingzhou, Hubei, China; ^3^ Jingzhou Rongjun Special Care Hospital, Jingzhou, Hubei, China; ^4^ Mental Health Center, West China Hospital, Sichuan University, Chengdu, Sichuan, China; ^5^ Jingzhou Mental Health Center, Jingzhou, Hubei, China; ^6^ Institute of Psychiatry, West China Hospital, Sichuan University, Chengdu, Sichuan, China

**Keywords:** COVID-19, non-suicidal self-injury, youth students, mediation analysis, China

## Abstract

**Objective:**

This study aimed to explore the impact of the COVID-19 pandemic on non-suicidal self-injury (NSSI) among youth students, and the mediating role of psychological factors in the relationship between the COVID-19 pandemic and NSSI.

**Method:**

An online survey was conducted at junior and senior high schools, as well as universities located in Jingzhou, Hubei Province, China between June 2021 and January 2022. The COVID-19 Impact Index was constructed using multiple correspondence analysis (MCA) method. The bootstrapping method was used for mediation analysis.

**Results:**

A total of 16025 youth participated in the study and 12507 youth (78.1%) finished the questionnaires. The COVID-19 Impact Index had a significantly positive effect on NSSI (r=0.16, p<0.001). The mediation analysis results showed that the COVID-19 Impact Index had a significant indirect effect on youth’ NSSI (β=0.0918, 95% CI [0.0788, 0.1048]), and this indirect effect was mainly achieved through affecting youth’ anxiety, depression and post-traumatic stress disorder (PTSD). The mediation effect of anxiety on NSSI was 0.0584, the direct effect was 0.0334, and the mediation proportion was 63.6%. The mediation effect of depression on NSSI was 0.0668, the direct effect was 0.0250, and the mediation proportion was 72.8%. The mediation effect of PTSD on NSSI was 0.0640, the direct effect was 0.0278, and the mediation proportion was 69.7%. All the mediation effects, direct effects and total effects were statistically significant (p<0.001).

**Conclusion:**

The higher the impact of the COVID-19 Impact Index, the higher the prevalence of NSSI among youth students. Anxiety, depression and PTSD had mediated the relationship between the COVID-19 Impact Index and NSSI. It is suggested that specific health policies, mental health services and interventions should be developed to reduce the NSSI and improve mental health status among youth students during the COVID-19 pandemic.

## 1 Introduction

Non-suicidal self-injury (NSSI) has become a global public health concern, particularly among youth. NSSI refers to the behavior of intentionally causing physical harm to oneself without suicidal intent (1, 2). Research indicates that NSSI is not only a significant warning signal for suicidal behavior in youth but also a strong predictor of future suicidal actions (3). The onset age for NSSI in youth typically ranges from 12 to 14 years, peaking between 15 and 16 years, and gradually declining around 18 years (4). The prevalence and severity of NSSI among youth have garnered widespread attention, with lifetime prevalence rates ranging from 17.8% to 22.1% globally, encompassing various forms such as cutting, scratching, and burning (5–7). The etiology of NSSI is multifactorial, involving individual psychological traits, social environment, and family background (8). According to Nock’s behavioral model, NSSI is viewed as a means of regulating negative emotions or coping with stress (9). Further exploration reveals that individuals may engage in NSSI to escape negative emotions, self-punish, or seek attention and help from others (10). This behavior reflects coping mechanisms for dealing with internal pain and stress, highlighting deep-seated psychological needs and unmet emotional demands.

Depressive and anxiety disorders are common comorbidities among youth with NSSI (11–14). There is a significant bidirectional relationship between anxiety, depression, and NSSI: higher scores of anxiety and depression increase the risk of NSSI (15), and individuals who engage in NSSI often exhibit elevated levels of anxiety and depression (16). Meta-analytic evidence also underscores the significant impact of depression on NSSI (17, 18). For instance, a meta-analysis by Hawton et al. (19) shows that depression significantly predicts NSSI, with individuals experiencing higher levels of depressive symptoms being more likely to engage in self-injury. Additionally, the positive association between anxiety and NSSI has been confirmed by many previous studies (20). For example, a study by Glenn and Klonsky (21) indicates that severe anxiety is a crucial trigger for NSSI, as individuals with high anxiety levels are more prone to use self-injury as a coping mechanism to alleviate their distress (22).

Patients with post-traumatic stress disorder (PTSD) are at a higher risk of developing NSSI (23), especially when PTSD symptoms are severe (24, 25). Various symptom clusters of PTSD are considered as risk factors for NSSI (13), likely due to the emotional regulation difficulties these symptoms cause. For instance, after experiencing traumatic events, individuals may exhibit persistent avoidance behaviors, hyperarousal, and complex negative emotional changes, all of which can increase the likelihood of NSSI (26). Therefore, the emotional regulation difficulties associated with PTSD may play an important role in understanding the mechanisms underlying NSSI behavior.

The COVID-19 pandemic, which began at the end of 2019, has had profound impacts on the lives and mental health of people worldwide. Youth have faced prolonged isolation, changes in learning modes, and restrictions on social activities, significantly increasing their stress and challenges, leading to a marked rise in mental health issues (27–31). Studies have shown that, compared to the pre-pandemic period, the prevalence of depressive and anxiety symptoms among Chinese youth has significantly increased during and post-pandemic (32–34). This is particularly evident among college students, where levels of anxiety and depression have risen. Furthermore, research has found a close association between anxiety, depression, and the occurrence of NSSI (35, 36). During the pandemic, the incidence of NSSI among youth increased due to heightened academic pressure, social barriers, and family conflicts (37–40). Stress resulting from adverse and negative life events is a recognized risk factor for the occurrence and maintenance of NSSI (41, 42). Self-isolation and quarantine measures implemented by many countries have disrupted daily activities and habits, potentially leading to increased loneliness, anxiety, depression, insomnia, substance abuse, self-harm, or suicidal behaviors (43). While previous studies have extensively examined the general impact of the COVID-19 pandemic on mental health, there is a notable gap in the literature regarding the specific mechanisms through which pandemic-related stressors influence NSSI among youth. Most existing research has focused on adult populations or has not adequately addressed the mediating role of psychological factors such as anxiety, depression, and PTSD in the context of NSSI (36, 41–43). Therefore, it is crucial to explore the further impact of the COVID-19 pandemic on youth mental health and NSSI for developing effective prevention and intervention measures.

### 1.1 Theoretical framework

The Traumatic Stress Theory and the Stress-Vulnerability Model provide essential perspectives for understanding the mechanisms underlying NSSI among youths during the COVID-19 pandemic. Traumatic Stress Theory posits that exposure to traumatic events, such as the COVID-19 pandemic, can lead to complex emotional and behavioral responses, including anxiety, depression, and PTSD (44). These responses may drive individuals to engage in NSSI as a means of self-regulation. The pandemic represents a significant traumatic event that has disrupted the lives of youth, leading to heightened psychological distress. This theory helps explain why youth might resort to NSSI to cope with the overwhelming stress and emotional turmoil caused by the pandemic (9).

Previous studies have applied Traumatic Stress Theory to various contexts, demonstrating its relevance in understanding psychological responses to traumatic events. For instance, research has shown that traumatic experiences, such as natural disasters and personal assaults, can lead to increased rates of anxiety, depression, and PTSD, which in turn may result in maladaptive behaviors like NSSI (45, 46).

The Stress-Vulnerability Model suggests that an individual’s response to stress is influenced by their inherent vulnerabilities, such as genetic predisposition, early life experiences, and psychological traits (47, 48). According to this model, the COVID-19 pandemic acts as a significant stressor that interacts with these vulnerabilities, increasing the likelihood of maladaptive coping mechanisms like NSSI. This model is particularly relevant to this study as it highlights the interplay between external stressors (e.g., pandemic-related disruptions) and internal vulnerabilities (e.g., pre-existing mental health conditions) (49). The Stress-Vulnerability Model has been extensively applied in mental health research to explain how stress and individual vulnerabilities contribute to various psychological disorders. For example, studies have shown that individuals with a genetic predisposition to mental health issues are more likely to develop conditions such as anxiety and depression when exposed to significant stressors (50, 51).

By integrating these theories, we can better understand the multifaceted impact of the COVID-19 pandemic on youth mental health and NSSI. The Traumatic Stress Theory elucidates how the pandemic acts as a traumatic event leading to psychological distress, while the Stress-Vulnerability Model explains how individual vulnerabilities interact with this stress to result in NSSI. Together, these theories offer a cohesive framework for examining the direct and indirect effects of pandemic-related factors on NSSI, mediated by psychological factors such as anxiety, depression, and PTSD.

### 1.2 Research objectives

Based on the Traumatic Stress Theory and the Stress-Vulnerability Model, this cross-sectional study aimed to explore the psychological causes of NSSI among youth students during the COVID-19 pandemic and its associations with anxiety, depression, and PTSD. The specific research objectives include: 1) Assessing the impact of the COVID-19 pandemic on the prevalence of NSSI among youth students, and establishing the COVID-19 Impact Index to quantify and analyze the various dimensions of the pandemic’s influence on the population; and 2) Examining the mediating effects of anxiety, depression, and PTSD symptoms in the relationship between the COVID-19 pandemic and NSSI.

### 1.3 Research hypotheses

Based on the Traumatic Stress Theory and the Stress-Vulnerability Model, the following hypotheses are proposed: Hypothesis 1 (H1): There is a positive relationship between COVID-19 pandemic impact factors and NSSI behavior among youth students. Hypothesis 2 (H2): Anxiety, depression, and PTSD respectively mediate the relationship between COVID-19 pandemic impact factors and NSSI.

## 2 Methods and materials

### 2.1 Participants and procedure

This study was a cross-sectional online survey using a cluster sampling method in the urban area of Jingzhou, Hubei Province, China, from June 2021 to January 2022. After contacting the schools, eight schools agreed to participate in the survey, including all their students: three junior high schools, three senior high schools, and two universities. The researchers and teachers involved in the survey were trained first, and then the questionnaires were filled out by students in these schools or universities after obtaining their informed consent. The youth students completed the questionnaire via WeChat scanning code, and trained researchers answered students’ questions throughout the survey process. This study was approved by the Ethics Committee of Yangtze University, with ethics approval number 2021LL0501.

### 2.2 Measurements

#### 2.2.1 Self-developed general information questionnaire

General demographic information and the COVID-19 pandemic information were collected. The demographic information included grade, gender, age, family economic status, only-child status, family structure, and etc. The pandemic-related information included quarantine status, psychological stress levels during the COVID-19 period, family and friend relationships due to the pandemic, and the impact of COVID-19 pandemic on educational progress and post-epidemic recovery in education and daily life. Quarantine status was defined as either medical or centralized quarantine. Psychological stress was rated on a scale from 1 to 10, with a median score of 5 serving as the threshold; scores of ≤5 indicating low psychological stress, while scores of >5 denoting high psychological stress. The selection of these COVID-19 variables was based on a combination of previous literature and preliminary analysis. Previous studies have highlighted the significant impact of quarantine status, psychological stress, and changes in family and social relationships on mental health during the pandemic. For instance, a study by Wang et al. (52) found that quarantine measures were associated with increased risk of psychological outcomes, particularly among vulnerable groups. Another systematic review by Bonati et al. (53) demonstrated that anxiety, depression, and post-traumatic symptoms were frequently experienced during quarantine, often linked to changes in sleeping and eating habits. Additionally, research showed that work-family conflict and increased parental concerns during the pandemic were associated with higher levels of depressive symptoms (54). Preliminary analysis indicated that these variables might be relevant and significant in the context of this study population. Therefore, these variables were chosen to comprehensively capture the multifaceted impact of the COVID-19 pandemic on youth students.

#### 2.2.2 Adolescent self-injury behavior questionnaire 

The ASIB has 12 items (55), including self-harm frequency and severity. The scale had good reliability and validity (56), Cronbach’s alpha for this questionnaire was 0.88. This study only involved the self-harm frequency part, which has four levels: 0, 1, 2-4, ≥5 times. According to the Diagnostic and Statistical Manual of Mental Disorders, Fifth Edition (DSM-V), a total frequency of NSSI ≥5 times is classified as “frequent self-injury,” 1-4 times as “occasional self-injury,” and 0 times as “no self-injury” (57). This study used NSSI frequency as an indicator of NSSI behavior, and self-harm frequency ≥1 indicated the presence of NSSI.

#### 2.2.3 Generalized anxiety disorder-7

The scale was developed by Spitzer RL et al. with 7 questions, covering anxiety symptoms that occurred in the past two weeks, and was commonly used in mental health area (58). Each item is rated on a 4-point scale from 0 to 3 according to the frequency of occurrence in the past two weeks, with a total score range of 0-21. The higher the total score, the more severe the anxiety level.

#### 2.2.4 Patient health questionnaire

The scale was developed by K Kroenke et al. (9) with 9 items, covering the core symptoms of depression, such as low mood, loss of interest, suicidal ideation, etc., and was commonly used to assess the depression level (59). Each item is rated on a 4-point scale from 0 to 3 according to the frequency of occurrence in the past two weeks, with a total score range of 0-27. The higher the total score, the more severe the depression level.

#### 2.2.5 Posttraumatic stress disorder checklist for DSM‐5

The scale was developed by Weathers, F.W et al. with 20 questions, covering the symptoms of post-traumatic stress disorder (PTSD), such as re-experiencing, avoidance, negative alterations in cognition and mood, and hyperarousal, and was commonly used to assess the PTSD (60). Each item is rated on a 5-point scale from 0 to 4 according to the frequency of occurrence in the past month, with a total score range of 0-80. The higher the total score, the more severe the PTSD level.

### 2.3 Statistical analysis

#### 2.3.1 Multiple correspondence analysis

Using the FactoMineR package, MCA was conducted to handle large data matrices containing binary, ordinal, or nominal variables (61). MCA simplifies complex data into a few orthogonal dimensions, revealing relationships between variables that pairwise analysis cannot detect. In this study, MCA was used to assess relationships among all features and to evaluate the complex associations between 10 pandemic-related variables (e.g., Isolation status, Psychological stress level during the COVID-19 epidemic period, Family economic status affected by the COVID-19 epidemic, Family relationships affected by the COVID-19 epidemic, etc.) and youth NSSI. To interpret MCA results, we referred to inertia, eigenvalues, contributions, and factor coordinates. Additionally, heatmaps were created to help interpret MCA, using color intensity to show the level of association between variables, thus displaying associations through distances between categories in the MCA plot (62).

#### 2.3.2 Propensity score matching

PSM was used to address potential confounding factors between the NSSI and non-NSSI groups, enhancing causal inference accuracy. First, propensity scores for each participant were calculated using a logistic regression model that included all known confounding variables, such as grade, gender, who do you currently live with, and only child. Based on these scores, individuals in the NSSI group were matched with those in the non-NSSI group to ensure comparability on key variables. The matching process was completed using the MatchIt package in R (63), and matching quality was assessed using standardized differences. By comparing the matched groups on primary outcomes, we estimated the causal effect of the COVID-19 pandemic on youth NSSI behavior. This method might reduce selection bias in observational studies and improved the validity of statistical inferences.

#### 2.3.3 Correlation analysis

Pearson correlation coefficients between variables were calculated using the cor function, and the correlation matrix was visualized using the corrplot package (64).

#### 2.3.4 Binary logistic regression

The glm function was used to analyze the direct impact of the COVID-19 pandemic on youth NSSI behavior and the moderating effects of negative coping styles and emotional regulation abilities (65).

#### 2.3.5 Mediation analysis

The mediation package was used to analyze the mediating role of PHQ-9, GAD-7, and PCL-5 scores in the relationship between the COVID-19 pandemic and NSSI behavior (17, 66–69).

## 3 Results

### 3.1 Characteristics of participants

A total of 16025 questionnaires were collected, and 12507 valid data (78.1%) were included in the study. Among participants in the study, there were 5326 (42.6%) males and 7181 (57.4%) females; 2839 (22.7%) were junior high school students, 2421 (19.4%) were high school students, and 7247 (57.9%) were college students; the average age of middle school students was 14.09 ± 0.94 years, high school students was 17.16 ± 1.12 years, and university students was 20.25 ± 1.21 years. The rate of NSSI was 19.4% (2425/12507). **Table 1** shows the distribution of the NSSI group and the self-harm group in various groups. There were significantly statistical differences between the two groups in terms of age, gender, grade, living situation, only-child status, quarantine status, psychological stress level during the COVID-19 pandemic, family economic status, family relationship, friend relationship, impact of the pandemic on further education, learning and life, recovery of learning and life after the pandemic (P<0.01).

**Table 1:** Characteristics and Variable Distribution of the Matched Samples.

Variable	NSSI group n (%)	Non-NSSI n (%)	χ^2^/Z/t	p-value
Grade				
Junior High School	2202(77.56)	637(22.44)	152.155	<0.001
Senior High School	1781(73.56)	640(26.44)		
College	6099(84.16)	1148(15.84)		
Gender				
Male	4356(81.79)	970(18.21)	8.086	0.004
Female	5726(79.74)	1455(20.26)		
Age (years)	18.37 (± 2.8)	17.79 (± 2.7)	9.5139	<0.001
Who do you currently live with				
Living with parents	7002(82.01)	1536(17.99)	69.313	<0.001
Living with grandparents or both parents	1975(80.45)	480(19.55)		
Single-parent family (divorced parents)	482(72.16)	186(27.84)		
Reconstituted family (remarried parents)	202(71.13)	82(28.87)		
Other	421(74.91)	141(25.09)		
Only child				
No	6326(81.47)	1439(18.53)	9.486	0.002
Yes	3756(79.21)	986(20.79)		
Isolation status				
Yes	1027(75.91)	326(24.09)	21.156	<0.001
No	9055(81.18)	2099(18.82)		
Psychological stress level during the COVID-19 epidemic period				
Low level	8710(81.69)	1952(18.31)	53.583	<0.001
High level	1372(74.36)	473(25.64)		
Family economic status affected by the COVID-19 epidemic				
Yes	3697(78.43)	1017(21.57)	22.883	<0.001
No	6385(81.93)	1408(18.07)		
Family relationships affected by the COVID-19 pandemic				
Yes	744(66.13)	381(33.87)	164.760	<0.001
No	9338(82.04)	2044(17.96)		
Friendships affected by the COVID-19 pandemic				
Yes	744(66.13)	381(33.87)	126.767	<0.001
No	9398(81.80)	2091(18.20)		
Impact of the COVID-19 pandemic on education advancement				
Yes	6244(79.01)	1659(20.99)	35.013	<0.001
No	3838(83.36)	766(16.64)		
Post-pandemic education recovery				
Yes	4876(85.13)	852(14.87)	137.291	<0.001
No	5206(76.80)	1573(23.20)		
Post-pandemic life recovery				
Yes	5262(83.67)	1027(16.33)	75.343	<0.001
No	4820(77.52)	1398(22.48)		
Impact of the COVID-19 pandemic on education				
Yes	3402(76.12)	1067(23.88)	89.104	<0.001
No	6680(83.11)	1358(16.89)		
Impact of the COVID-19 pandemic on life				
Yes	726(68.30)	337(31.70)	111.841	<0.001
No	9356(81.75)	2088(18.25)		
GAD-7 Score	0(0~3)	5(1~8)	-43.951	<0.001
PHQ-9 Score	0(0~4)	7(3~11)	-44.587	<0.001
PCL-5 Score	0(0~6)	14(5~24)	-45.631	<0.001

### 3.2 The COVID-19 impact index

The study applied MCA to ten variables related to the pandemic within the questionnaire to examine their association with youth NSSI. These variables included quarantine status, psychological stress level during the COVID-19 pandemic, family relationships, friendships, and the impact of the pandemic on educational progression, recovery post-pandemic in education and life, as well as the overall effect of the pandemic on education and life. The MCA was used to reduce the dimensionality of the data and identify key dimensions that represent the underlying structure of the pandemic’s impact on youth students. The analysis revealed two primary dimensions, accounting for 35.1% of the total inertia. The first dimension accounted for 23.1% of the variance and primarily reflected youth students’ adaptability during and after the pandemic. The second dimension explained 12.0% of the variance and mainly reflected the extent of the pandemic’s impact on youth psychological, academic, and daily life aspects.

To construct the COVID-19 Impact Index, we combined the scores from these two dimensions. Each variable’s contribution to the dimensions was weighted based on its loading, reflecting its importance in explaining the variance. The index was calculated by summing the weighted scores of the two dimensions for each participant. This approach allowed us to create a composite score that captures the multifaceted impact of the pandemic on youth, considering both their adaptability and the overall effect on their lives.

**Figure 1:**
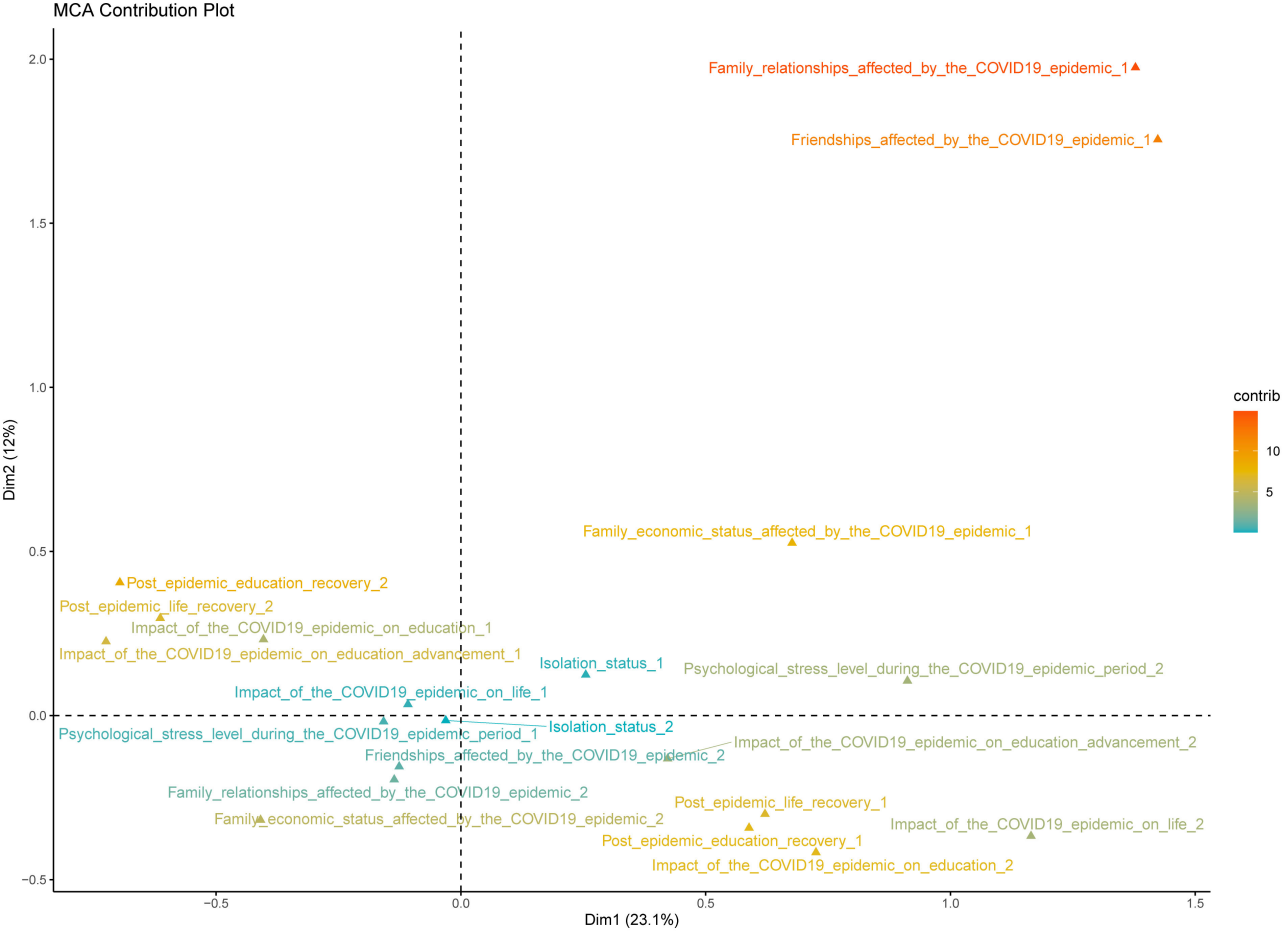
MCA Contribution Plot.

**Figure 1** illustrates the contributions of 10 categorical variables across the two dimensions. The first dimension might be closely related to youth resilience and coping strategies, indicating their psychological and behavioral adjustments in response to pandemic challenges. The second dimension might be associated with youth mental health and well-being, highlighting the importance of social support, family, and peer relationships for individual psychological health.

**Figure 2:**
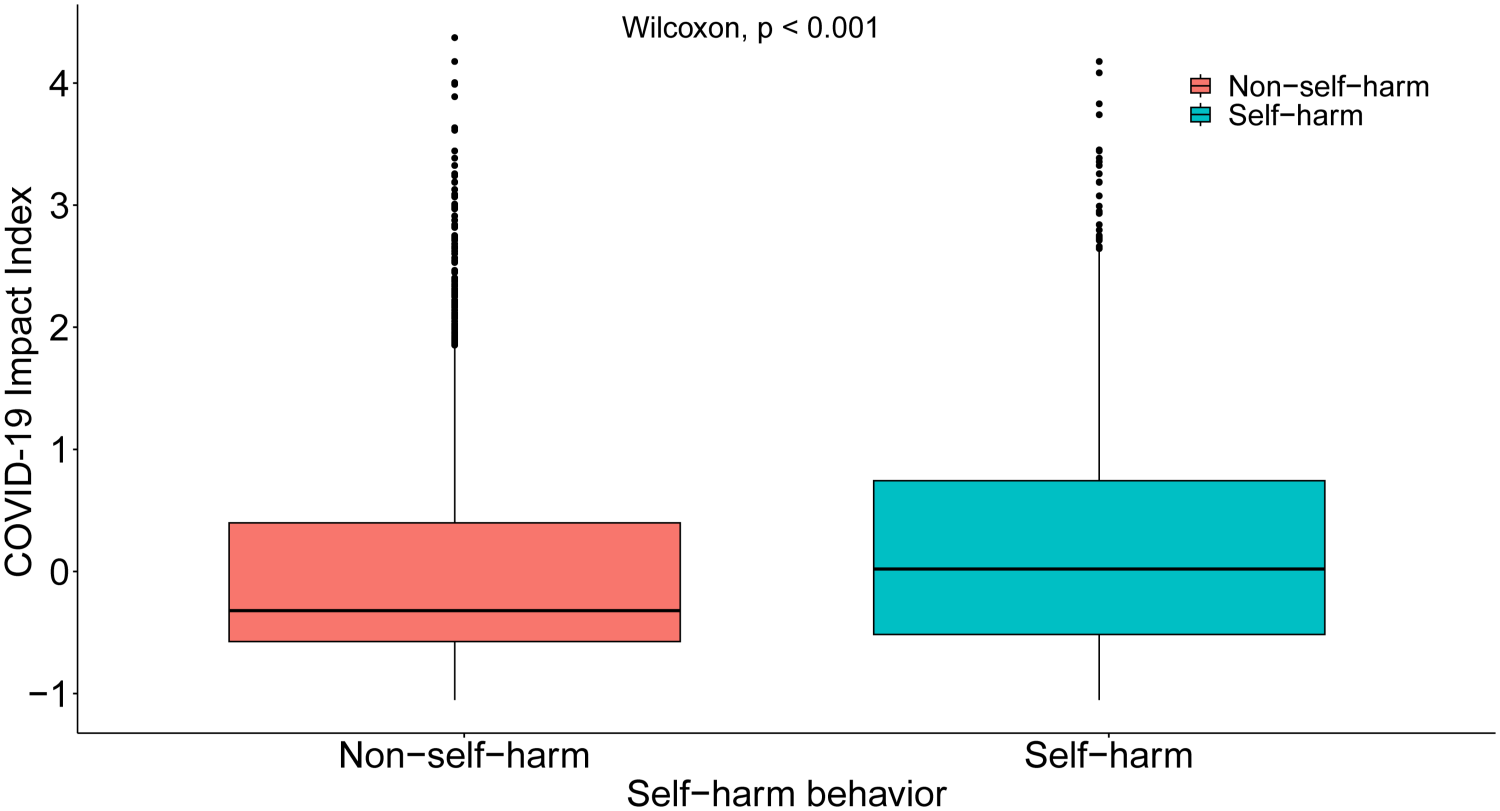
Comparison of NSSI and COVID-19 Impact Index: Boxplot Analysis.

**Figure 2** presents the differences between the self-harm group and the NSSI group on the COVID-19 Impact Index. The findings indicated that the mean value of the self-harm group on the COVID-19 Impact Index was significantly higher than that of the NSSI group (p<0.001). These outcomes highlighted a complex and multifaceted relationship between COVID-19-related factors and youth NSSI, necessitating a multi-angle and multi-level analysis and understanding.

### 3.3 Basic characteristics and variable distribution after matching

To reduce potential confounding factors between the self-injury group and the non-self-injury group, we employed the propensity score matching (PSM) method. PSM aims to minimize differences between the two groups on unobserved covariates by matching individuals from both groups. In this study, we used a logistic regression model to calculate the propensity scores for each participant based on covariates such as age, gender, family composition, and only-child status. These scores might reflect the conditional probability of each youth engaging in self-injury given the covariates.

Generation of Propensity Scores: We first identified key covariates that might influence youth NSSI and included these variables in the logistic regression model. The model output was the propensity score, representing the probability of each youth belonging to the self-injury group given the covariates. These scores were then used for the matching process.

After matching, we conducted balance tests to ensure that the matching process successfully balanced the distribution of covariates between the two groups. We used standardized differences and t-tests to assess the balance of covariates before and after matching. The standardized differences for the covariates were as follows: Distance: 0.0027; Grade: 0.0054; Gender: 0.0004; Who do you currently live with: 0.0070; and Only-child: 0.0123. All standardized differences were less than 0.02, well below the conventional threshold of 10%, indicating that the matched two groups achieved good balance on these covariates.

Comparison of Variables Before and After Matching: The median COVID-19 Impact Index for non-self-harm was -0.32 (-0.59 - 0.40) before matching and -0.34 (-0.52 - 0.38) after matching. The median PCL-5 score was 0 (0 - 6) before matching and 1 (0 - 6) after matching. The median scores for GAD-7 score and PHQ-9 score remained unchanged. Before matching, there were significant differences between the two groups on these variables. After matching, these differences were significantly reduced, indicating good matching effectiveness. The matched sample included 2,425 youth in the self-injury group and 4,850 youth in the non-self-injury group, totally 7,275 youth.

### 3.4 Correlations between COVID-19 impact index, any self injury, GAD-7 score, PHQ-9 score, and PCL-5 score

To explore the interrelationships between COVID-19-related factors, mental health status, and youth NSSI, we conducted a detailed correlation analysis. By calculating Pearson’s correlation coefficients, we assessed the linear relationships between variables. In this study, all correlation tests were conducted using two-tailed t-tests, and all p-values were less than 0.001, indicating high statistical significance.

**Figure 3:**
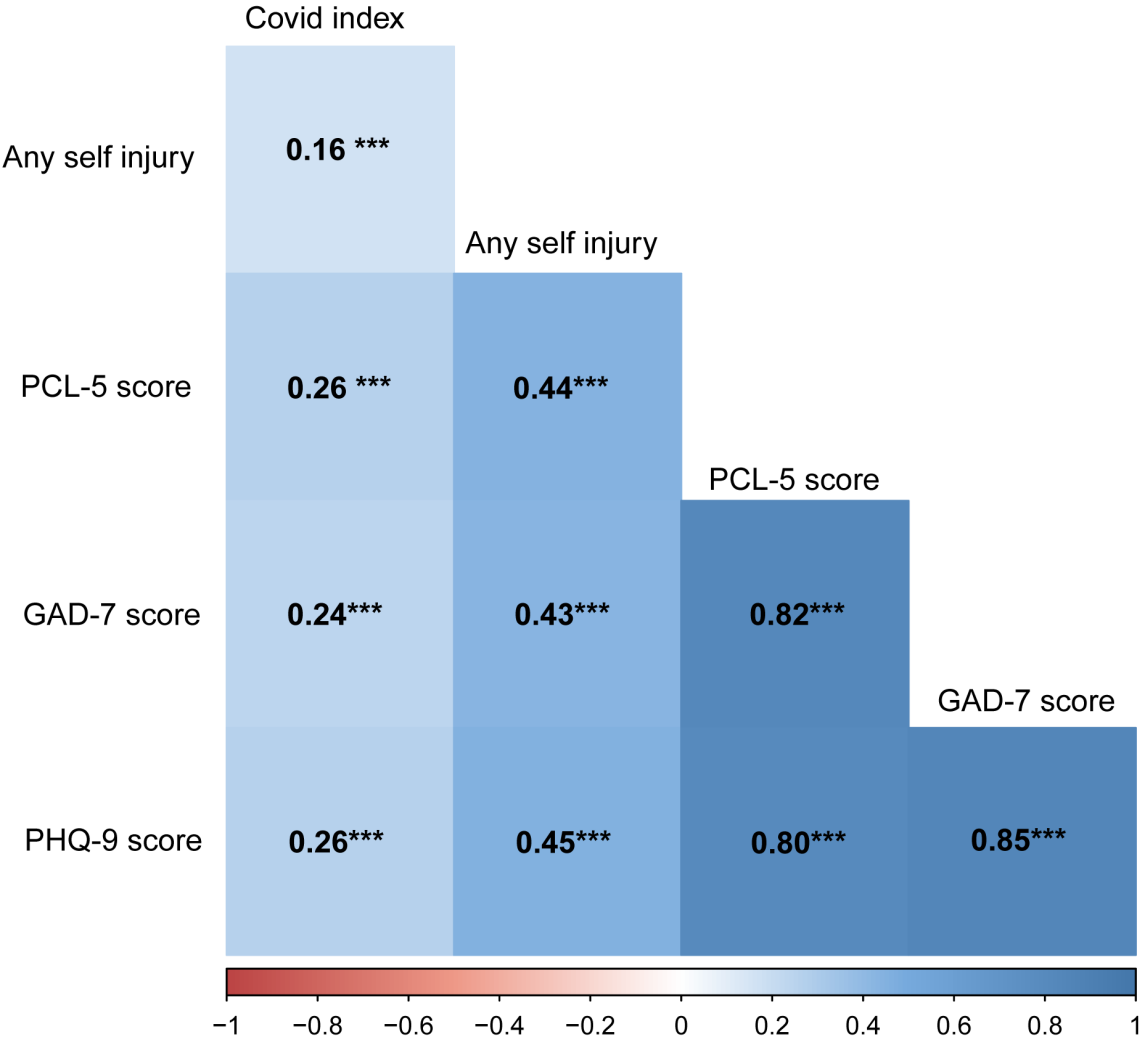
Correlation Matrix of Variables: COVID-19 Impact Index, Any Self Injury, GAD-7 Score, PHQ-9 Score, and PCL-5 Score. ***P<0.001.

Specifically, the correlation coefficient between the COVID-19 impact index and NSSI among youth was 0.16 (**Figure 3**). Additionally, the correlation coefficients between psychological factors such as anxiety, depression, and PTSD and NSSI were 0.43, 0.45, and 0.44, respectively, showing moderate to strong positive correlations. These data might emphasize the crucial role of mental health issues in youth NSSI, especially under the stress of the COVID-19 pandemic.

### 3.5 The influencing factors of NSSI

The binary logistic regression was used to evaluate the impact of the COVID-19 Impact Index, anxiety, depression, and PTSD on NSSI among participants. The results showed that all four variables had a statistically significant positive correlation with NSSI, indicating that students who were more affected by the COVID-19 pandemic, with higher levels of anxiety, depression, and PTSD were more likely to engage in NSSI (**Table 2**). The Nagelkerke R-squared value was 0.29, which might indicate that the variables in the model provided a considerable degree of explanation for predicting NSSI.

**Table 2:** Binary Logistic Regression Results.

Variable	OR ^#^	95% CI	p-value
COVID-19 Impact Index	1.106	1.033, 1.186	0.004
GAD7 score	1.036	1.011, 1.063	0.006
PHQ9 score	1.115	1.092, 1.139	0.000
PCL5 score	1.041	1.032, 1.049	0.000

^#^OR, Odds Ratio evaluated on the standardized values. The reference group is NO-NSSI

### 3.6 Mediation analysis: the role of GAD-7, PHQ-9, and PCL-5 scores as mediators

**Figure 4:**
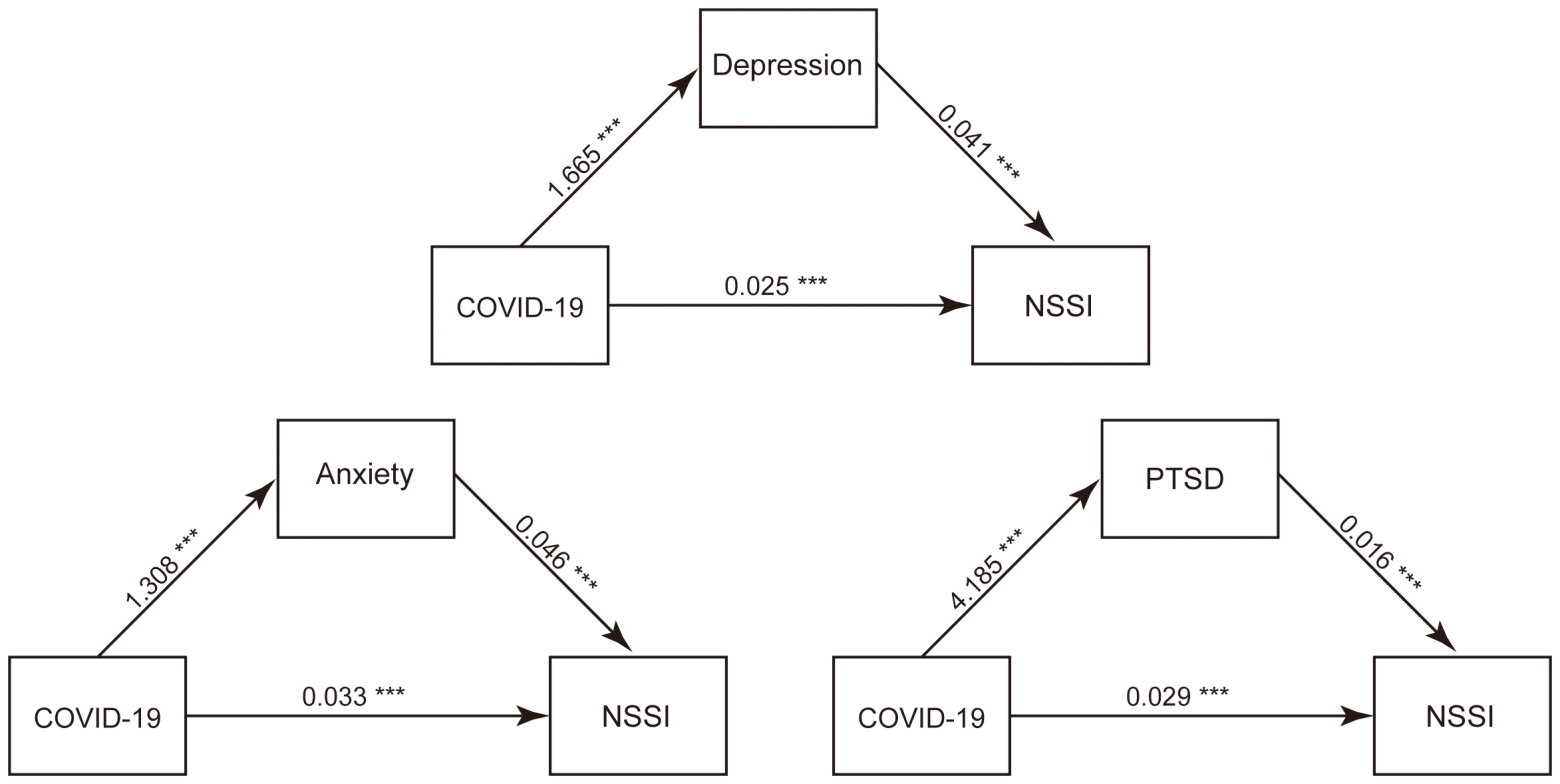
Mediation Effects Plot: COVID-19 Impact Index on NSSI through GAD-7 Score, PHQ-9 Score, and PCL-5 Score. ***P<0.001.

The GAD-7, PHQ-9, and PCL-5 Scores were employed as mediator variables, the “COVID-19 Impact Index” as the independent variable, and NSSI as the outcome variable. Regression models were built utilizing the lm function to investigate the linkages between the independent variable and the mediator variables, and also among the independent variable, mediator variables, and the dependent variable. The mediate function was used for mediation analysis. The visualization of mediation effects is illustrated in **Figure 4**, and the corresponding quantitative results are presented in **Table 3**. The findings revealed that all three measures, including GAD-7 Score, PHQ-9 Score, and PCL-5 Score, exerted significant mediating effects between the COVID-19 Impact Index and NSSI. This might suggest that the impact factor associated with the pandemic increased the likelihood of NSSI occurrence by elevating levels of anxiety, depression, and PTSD.

**Table 3:** Mediation Analysis Results: Pathways and Indirect Effects of COVID-19 Impact Index on NSSI through GAD-7 Score, PHQ-9 Score, and PCL-5 Score.

Pathways	ME	ER	95 CI	
			LL	UL
Total indirect effect	0.0918	/	0.0791	0.1055
COVID-19 Impact Index→GAD-7 score→NSSI	0.0584	0.6359	0.0518	0.0652
COVID-19 Impact Index→PHQ9_score→NSSI	0.0668	0.7278	0.0597	0.0740
COVID-19 Impact Index→PCL5_score→NSSI	0.0640	0.6971	0.0567	0.0719

ME, mediating effect; ER, effect ratio; CI, confidence interval; LL, lower level; UL, upper level.

## 4 Discussion

The aim of this study was to assess the impact of COVID-19-related factors on youth NSSI and to explore the potential mediating psychological mechanisms. Based on the Traumatic Stress Theory and the Stress-Vulnerability Model, this study conducted an online survey of youth students in Jingzhou, Hubei Province, China, from June 2021 to January 2022. Using MCA, the study comprehensively analyzed 10 variables related to the impact of the pandemic on youth, constructing a “COVID-19 Impact Index” to evaluate the impact extent of COVID-19-related factors on youth and their adaptability during the pandemic. Subsequently, correlation analysis, binary logistic regression, and mediation analysis were employed to thoroughly examine the impact of COVID-19-related factors on youth NSSI and the mediating role of psychological factors.

This study showed an overall NSSI detection rate of 19.4%, which is consistent with some studies both nationally and internationally (70–72). For example, a study reported a global prevalence of NSSI among adolescents ranging from 17.2% to 28.9%, aligning with our findings (40). Demographically, significant differences were found between the self-injury and non-self-injury groups in terms of age, gender, grade, and whom currently live with, indicating different distribution patterns of NSSI among various population characteristics. Regarding grade distribution, this study showed that the NSSI rate was 22.44% among junior high school students, 26.44% among senior high school students, and 15.84% among university students, with the highest detection rate among senior high school students. Systematic reviews also indicate that the detection rate of NSSI peaks in mid-adolescence (around 15-16 years) and declines in late adolescence (around 18 years) (11, 73). This is consistent with findings from Groschwitz et al. (74), which showed similar age-related trends in NSSI prevalence. Youth is a high-risk period for self-injury due to increased impulsivity and emotional reactivity resulting from brain development, making youth more prone to self-injury. During this period, emotional systems such as the amygdala mature earlier, while cognitive regulatory systems like the prefrontal cortex, which are involved in emotion regulation and decision-making, develop more slowly. This developmental mismatch increases the risk of risky and impulsive behaviors in youth (75, 76). This aligns with research by Romer et al. (77), which highlighted the developmental imbalance between emotional and cognitive regulatory systems during adolescence. Additionally, the proportion of females in the self-injury group was higher than that of males, and the overall psychological impact of the pandemic on females was greater. This may be related to fluctuations in ovarian hormone levels during specific stages of the menstrual cycle, leading to changes in sensitivity to emotional stimuli. This finding is consistent with studies by Lim and Tresno (6, 78), which indicated that females were more likely to engage in NSSI due to hormonal and emotional factors. Furthermore, the proportion of single-parent and reconstituted families was higher than that of two-parent families (79, 80), which is consistent with previous studies indicating the higher risk of NSSI among youth from incomplete or broken families (81). For example, a study by Coppersmith et al. (82) found that adolescents from single-parent or reconstituted families exhibited higher rates of NSSI compared to those from two-parent families.

This study showed a significant positive correlation between the COVID-19 Impact Index and youth NSSI, indicating that COVID-19, as an important environmental factor, has a direct or indirect impact on youth NSSI. Specifically, the COVID-19 Impact Index encompasses multiple dimensions such as quarantine status, psychological stress levels, family economic status, family relationships, friendships, education advancement, and recovery of learning and daily life, reflecting the extent of the pandemic’s impact on youth’ learning, life, and social interactions, as well as their adaptability.

Quarantine or isolation status, in particular, was significantly associated with youth self-injury behavior. While isolation measures help control virus transmission (83), prolonged isolation will increase social isolation and feelings of loneliness, potentially triggering widespread psychological stress responses such as loneliness, anxiety, and depression (84–86). These psychological stress responses may prompt youth students to engage in self-injury as a self-regulation mechanism. This finding is consistent with previous studies, such as Costa et al. (87) found that increased isolation during the pandemic was linked to higher levels of psychological distress and self-injury among adolescents.

Additionally, the results of this study showed that increased psychological stress levels during the pandemic, especially due to school closures, concerns about family members’ health, and drastic changes in daily life, significantly increased youth’ psychological stress, which is consistent with previous global studies (35, 88). For example, Zhang et al. (89) reported similar findings that school closures and family health concerns were major stressors contributing to increased anxiety and depression among youth. The deterioration of family economic status, particularly due to the economic recession caused by the pandemic, increased family tension and conflict, further affecting students’ mental health (90). Tense family relationships may weaken youth’ primary sources of emotional support, making them more likely to resort to self-injury to cope with negative emotions (91). This aligns with findings from a study by Magson et al. (92), which highlighted the impact of economic stress and family conflict on youth mental health during the pandemic. Changes in friendships, especially the reduction of face-to-face interactions with peers due to pandemic restrictions, were also significantly associated with youth self-injury behavior (93). The loss of peer support may make it more difficult for students to cope with pandemic-related stress, potentially leading to self-injury (36, 94). This is supported by research from Hou et al. (95), which found that reduced social interactions and peer support were significant predictors of increased self-injury behaviors among adolescents during the pandemic. The COVID-19 pandemic might significantly affect youth’ educational pathways. The pandemic forced school closures, requiring students to switch to online learning, which impacted their study habits and efficiency (95–97). This study found that students whose lives were more severely affected by the pandemic were more likely to engage in self-injury (98, 99). People’s lifestyles may be impacted for long term, even after the pandemic ends. Different population characteristics may reflect different trauma sensitivities or exposure levels, thereby affecting the incidence of NSSI (100–102).

COVID-19 pandemic, as a traumatic event, can lead to persistent and complex psychological stress responses in youth (103), such as anxiety, depression, and PTSD (104, 105), which can affect youth’ cognitive evaluation and emotional regulation abilities regarding traumatic events and their consequences, thereby increasing the risk of adverse outcomes such as NSSI (11, 73, 106–108), which supports the Traumatic Stress Theory and our hypothesis (H1). The results of this study showed that anxiety, as a psychological stress response, played a significant mediating role in students’ NSSI. Mediation analysis results also showed that the COVID-19 Impact Index had a significant indirect effect on NSSI through anxiety symptoms. This finding indicates that increased environmental stress during the pandemic may elevate youth’ anxiety levels, which in turn may lead to NSSI behavior. During the pandemic, youth may face multiple stressors such as school closures, social activity restrictions, and increased family economic pressure, all of which can lead to increased anxiety (109, 110). When youth feel unable to effectively cope with these stressors, they may resort to NSSI as a self-regulation method to alleviate internal tension and discomfort (39). Zhang et al. (111) also found that increased anxiety during the pandemic was linked to higher rates of NSSI among adolescents. To reduce NSSI behavior, targeted mental health support and interventions should be provided to help students better manage anxiety, develop effective coping strategies, and enhance their psychological resilience.

The results of this study showed that depression had a significant indirect effect between pandemic factors and self-injury, which is consistent with previous studies (112). The mediating effect of depression explained 72.8% of the total effect. Depression, as a negative emotional experience, can lead to a range of cognitive and behavioral impairments when severe, increasing the individual’s internal harm, such as suicidal ideation and behavior (113, 114). Additionally, the increase in depressive symptoms not only directly negatively impacts individuals’ daily functioning and quality of life but also indirectly affects their health by increasing the risk of NSSI behavior (39, 115). This finding underscores the importance of early identification and intervention for depressive symptoms in students during the pandemic to reduce the occurrence of NSSI behavior. Hu et al. (116) also reported that increased depressive symptoms during the pandemic were significantly associated with higher rates of NSSI among youth.

The results of this study showed that the mediating effect of PTSD symptoms explained 69.7% of the total effect between pandemic impact factors and NSSI. During the COVID-19 pandemic, many people experienced unprecedented stress and trauma, which may have led to increased PTSD symptoms (117). Our findings indicate that PTSD symptoms are an important mediating variable in the relationship between the pandemic and NSSI (102), emphasizing the importance of early identification and intervention for PTSD symptoms (e.g., psychosocial support and interventions) during the pandemic. Serafini et al. (118) also highlighted the role of PTSD symptoms in exacerbating NSSI behaviors during the pandemic.

In summary, this study established three mediation models, showing that GAD-7, PHQ-9, and PCL-5 scores all played significant mediating roles between pandemic impact factors and NSSI. These results verified the mediation model (H2). This indicates that pandemic impact factors increase the likelihood of NSSI by elevating levels of anxiety, depression, and PTSD. These findings support the Traumatic Stress Theory and the Stress-Vulnerability Model, suggesting that the COVID-19 pandemic not only directly increase youth self-injury behavior but also indirectly increased the risk of self-injury by affecting youth’ mental health. Our findings reveal how pandemic-related stress influences NSSI behavior through psychological factors such as anxiety, depression, and PTSD, echoing international research and emphasizing the importance of understanding and addressing student mental health issues globally (119–121). This study provides valuable data and insights for understanding and preventing students’ NSSI, helping to identify high-risk groups significantly affected by the pandemic, and offering effective psychological interventions and support. The study also offers practical and policy recommendations, such as enhancing attention and support for students in learning, life, and social aspects, improving their cognitive evaluation and emotional regulation abilities regarding the pandemic and its consequences, and strengthening their coping strategies and resources to promote their return to normal learning and life in the new normal.

This study has made theoretical contributions to the understanding of NSSI among youth students during the COVID-19 pandemic. By integrating the Traumatic Stress Theory and the Stress-Vulnerability Model, this research provides a comprehensive framework for examining the multifaceted impact of the pandemic on youth mental health and NSSI behavior. The Traumatic Stress Theory extends its application by demonstrating how a global traumatic event, such as the COVID-19 pandemic, can lead to increased psychological distress (anxiety, depression, PTSD) and subsequently elevate the risk of NSSI among youth. This highlights the importance of considering large-scale traumatic events in the context of youth mental health. The Stress-Vulnerability Model is supported by showing that individual vulnerabilities (e.g., pre-existing mental health conditions) interact with external stressors (e.g., pandemic-related disruptions) to influence NSSI behavior, underscoring the need to address both environmental stressors and individual vulnerabilities in interventions for reducing NSSI. By employing mediation analysis, this study elucidates the pathways through which pandemic-related factors influence NSSI, specifically through the mediating effects of anxiety, depression, and PTSD, providing a nuanced understanding of the mechanisms underlying NSSI and highlighting potential targets for psychological interventions.

The innovation of this study lies in the use of MCA, a multivariate analysis method, to transform 10 categorical variables into a single numerical variable—the “COVID-19 Impact Index”—thereby simplifying the data structure, improving data analysis efficiency, and allowing exploration of relationships between variables from multiple dimensions. The Pandemic Impact Factor serves as an indicator to measure the extent of the pandemic’s impact on students’ social, environmental, and interpersonal aspects, help to develop effective coping strategies, and assess the risks posed by the pandemic to students. The role and significance of the Pandemic Impact Factor are to provide an objective, quantifiable, and comparable indicator to reflect the complexity and multidimensionality of the pandemic, as well as the extent of its impact on youth students and their resilience in facing the pandemic. This study used the COVID-19 Impact Index to measure the extent of the pandemic’s impact on youth students’ lives and learning. However, MCA has some limitations and shortcomings, such as data quality, variable selection, and dimension selection, and it has not undergone rigorous reliability and validity testing. Further studies should be conducted to explore the reliability and validity of the COVID-19 Impact Index using MCA.

Nevertheless, this study also has some limitations and shortcomings. This study is a cross-sectional design. Thus, while correlation analysis, binary logistic regression, and mediation analysis methods were used to explore the relationships and mediating effects between variables, they cannot determine causal relationships or consider nonlinear relationships or interactions between variables. These limitations may affect the depth and breadth of the study’s findings. Therefore, future research could further deepen the understanding of youth students’ NSSI by using more rigorous experimental designs, more complex models, and more appropriate correlation coefficients.

## 5 Conclusion

This study adopted the trauma-stress theory and the stress-vulnerability model to assess the impact of COVID-19 related factors on youth’ NSSI, and explored the possible mediating role of psychological factors. The results of this study showed that there was a complex and diverse relationship between COVID-19 related factors and NSSI, and this relationship was mainly achieved by affecting anxiety, depression, and PTSD among youth students. This study provided valuable data and insights for understanding and preventing NSSI among youth students, as well as some suggestions and implications for mental health policies and practice. Further studies should be conducted in the important research area.

## Data Availability

The datasets presented in this article are not readily available because the dataset contains sensitive information related to adolescent non-suicidal self-injury behaviors and psychological factors. To protect the privacy and confidentiality of the participants, access to the dataset is restricted. Identifiable information has been anonymized, and any use of the dataset must comply with ethical guidelines and data protection regulations. Requests to access the datasets should be directed to BoLiu,drliubo2011@163.com.
